# Association of the Polygenic Scores for Personality Traits and Response to Selective Serotonin Reuptake Inhibitors in Patients with Major Depressive Disorder

**DOI:** 10.3389/fpsyt.2018.00065

**Published:** 2018-03-06

**Authors:** Azmeraw T. Amare, Klaus Oliver Schubert, Fasil Tekola-Ayele, Yi-Hsiang Hsu, Katrin Sangkuhl, Gregory Jenkins, Ryan M. Whaley, Poulami Barman, Anthony Batzler, Russ B. Altman, Volker Arolt, Jürgen Brockmöller, Chia-Hui Chen, Katharina Domschke, Daniel K. Hall-Flavin, Chen-Jee Hong, Ari Illi, Yuan Ji, Olli Kampman, Toshihiko Kinoshita, Esa Leinonen, Ying-Jay Liou, Taisei Mushiroda, Shinpei Nonen, Michelle K. Skime, Liewei Wang, Masaki Kato, Yu-Li Liu, Verayuth Praphanphoj, Julia C. Stingl, William V. Bobo, Shih-Jen Tsai, Michiaki Kubo, Teri E. Klein, Richard M. Weinshilboum, Joanna M. Biernacka, Bernhard T. Baune

**Affiliations:** ^1^Discipline of Psychiatry, School of Medicine, University of Adelaide, Adelaide, SA, Australia; ^2^Northern Adelaide Local Health Network, Mental Health Services, Adelaide, SA, Australia; ^3^Epidemiology Branch, Division of Intramural Population Health Research, National Institute of Child Health and Human Development, National Institutes of Health, Bethesda, MD, United States; ^4^HSL Institute for Aging Research, Harvard Medical School, Boston, MA, United States; ^5^Program for Quantitative Genomics, Harvard School of Public Health, Boston, MA, United States; ^6^Broad Institute of MIT and Harvard, Cambridge, MA, United States; ^7^Biomedical Data Science, Stanford University, Stanford, CA, United States; ^8^Department of Health Sciences Research, Mayo Clinic, Rochester, NY, United States; ^9^Department of Bioengineering, Stanford University, Stanford, CA, United States; ^10^Department of Psychiatry and Psychotherapy, University of Muenster, Muenster, Germany; ^11^Department of Clinical Pharmacology, University Göttingen, Göttingen, Germany; ^12^Department of Psychiatry, Taipei Medical University-Shuangho Hospital, New Taipei City, Taiwan; ^13^Department of Psychiatry and Psychotherapy, Faculty of Medicine, University of Freiburg, Freiburg, Germany; ^14^Department of Psychiatry and Psychology, Mayo Clinic, Rochester, NY, United States; ^15^Department of Psychiatry, Taipei Veterans General Hospital, Taipei, Taiwan; ^16^Division of Psychiatry, School of Medicine, National Yang-Ming University, Taipei, Taiwan; ^17^Department of Psychiatry, Faculty of Medicine and Life Sciences, University of Tampere, Tampere, Finland; ^18^Department of Molecular Pharmacology and Experimental Therapeutics, Mayo Clinic Rochester, Rochester, MN, United States; ^19^Department of Psychiatry, Seinäjoki Hospital District, Seinäjoki, Finland; ^20^Department of Neuropsychiatry, Kansai Medical University, Osaka, Japan; ^21^Department of Psychiatry, Tampere University Hospital, Tampere, Finland; ^22^RIKEN Center for Integrative Medical Sciences, Kanagawa, Japan; ^23^Department of Pharmacy, Hyogo University of Health Sciences, Hyogo, Japan; ^24^Center for Neuropsychiatric Research, National Health Research Institutes, Miaoli, Taiwan; ^25^Center for Medical Genetics Research, Rajanukul Institute, Department of Mental Health, Ministry of Public Health Bangkok, Bangkok, Thailand; ^26^Research Division Federal Institute for Drugs and Medical Devices, Bonn, Germany

**Keywords:** pharmacogenomics, polygenic score, personality traits, major depression, antidepressants, selective serotonin reuptake inhibitors

## Abstract

Studies reported a strong genetic correlation between the Big Five personality traits and major depressive disorder (MDD). Moreover, personality traits are thought to be associated with response to antidepressants treatment that might partly be mediated by genetic factors. In this study, we examined whether polygenic scores (PGSs) derived from the Big Five personality traits predict treatment response and remission in patients with MDD who were prescribed selective serotonin reuptake inhibitors (SSRIs). In addition, we performed meta-analyses of genome-wide association studies (GWASs) on these traits to identify genetic variants underpinning the cross-trait polygenic association. The PGS analysis was performed using data from two cohorts: the Pharmacogenomics Research Network Antidepressant Medication Pharmacogenomic Study (PGRN-AMPS, *n* = 529) and the International SSRI Pharmacogenomics Consortium (ISPC, *n* = 865). The cross-trait GWAS meta-analyses were conducted by combining GWAS summary statistics on SSRIs treatment outcome and on the personality traits. The results showed that the PGS for openness and neuroticism were associated with SSRIs treatment outcomes at *p* < 0.05 across P_T_ thresholds in both cohorts. A significant association was also found between the PGS for conscientiousness and SSRIs treatment response in the PGRN-AMPS sample. In the cross-trait GWAS meta-analyses, we identified eight loci associated with (a) SSRIs response and conscientiousness near *YEATS4* gene and (b) SSRI remission and neuroticism eight loci near *PRAG1, MSRA, XKR6, ELAVL2, PLXNC1, PLEKHM1*, and *BRUNOL4* genes. An assessment of a polygenic load for personality traits may assist in conjunction with clinical data to predict whether MDD patients might respond favorably to SSRIs.

## Introduction

A major depressive disorder (MDD) is the most common and disabling mental health diseases worldwide (1, 2) with a lifetime prevalence of ~12% (3). Studies estimated a 61.6 million years of life lived with disability caused by MDD accounting for 2.5% of the total disability-adjusted life years and for 8.1% of the total years lived with disability resulted from all diseases (2, 4).

Selective serotonin reuptake inhibitors (SSRIs) are commonly used as the first-line pharmacological treatment for MDD (5). However, treatment efficacy with SSRIs varies widely between individual patients and is inadequate in many cases. Clinical response rates range from 48 to 64% (6, 7) and reported remission rates are as low as 23.5% (7, 8). To improve this situation, an investigation of the biological and psychosocial factors that drive heterogeneity in treatment outcomes is necessary.

There is growing evidence from genetic studies that antidepressant treatment response is substantially influenced by genes (7, 9–17). A study involving nearly 3,000 MDD patients estimated that genetic factors explain 42% of the differences in the level of treatment response (18). A number of genes and single nucleotide polymorphisms (SNPs) that could influence antidepressant treatment outcomes have been reported, including polymorphisms within the *COMT* (9), *HTR2A* (10), *HTR1A* (11), *CNR1* (11), *SLC6A4* (12), *NPY* (13), *MAOA* (14), and *IL1B* (15) genes. A pharmacogenomic study on SSRIs response by the International SSRIs Pharmacogenomics Consortium (ISPC) identified several SNPs with suggestive association after 4 weeks of treatment, including the neuregulin-1 gene, which is involved in many aspects of brain development, such as neuronal maturation (7).

In addition to genetic factors, multiple demographic, clinical, and psychological predictors of SSRI response in MDD have been identified, collectively explaining 5–15% of the variance in treatment outcomes (19–23). Among the psychological predictors, personality traits defined by the Five-Factor Model of Personality (“Big Five”: extraversion, agreeableness, conscientiousness, neuroticism, openness) (24) have previously been reported to influence antidepressant treatment response and remission (25–29). Of these, neuroticism is a frequently reported predisposing factor for depression and was shown to negatively affect antidepressants treatment response (30, 31). In a recent study, MDD patients resistant to antidepressants were more likely to report high clinical scores for neuroticism, but low scores for openness, conscientiousness, and extraversion (26). In a large study of patients with MDD (*n* = 8,229), pre-existing personality dysfunction was associated with poor response to antidepressants (27). Further, some studies have suggested that SSRIs have a direct positive impact on scores for neuroticism or extraversion in MDD patients, and that part of the antidepressant effect might be explained through these adjustments (28, 29, 32, 33). Moreover, shared genes are thought to play a key role in the association between personality factors and MDD (34). For example, studies have estimated the genetic correlation between MDD and neuroticism at 55–75% (35, 36). However, no previous work has directly addressed the question whether there is a genetic relationship between the Big Five personality traits and SSRI treatment response and remission in MDD. It has been shown that the genetic architecture of personality traits is highly polygenic, in which several genes of small effect contribute to the overall phenotype (35, 37). Thus, a polygenic score (PGS) analysis approach proposed by the schizophrenia consortium (38) and later applied in several studies (16, 39), is potentially powerful to investigate the genetic influence of each of the Big Five personality traits on antidepressant treatment outcomes. A PGS for each of the Big five personality traits quantifies the combined effects of genetic variants across the whole genome, computed as a weighted summation of effect sizes obtained from genome-wide association studies (GWASs). A successful multi-trait polygenic model may assist for an early screening of diseases risk, clinical diagnosis, and the prediction of treatment response and prognosis (38, 39).

Implicitly, one could also interpret a polygenic association as a biological relationship partly explained by the role of shared genes and common molecular mechanisms. With this in mind, we conducted GWAS meta-analyses by combining GWAS summary statistics on the Big Five personality traits and SSRIs treatment outcome to identify shared genes involved in the cross-trait association.

## Materials and Methods

The characteristics of the clinical and genetic data, as well as the sources of the GWAS summary statistics used in our analysis are described below.

### Study Samples

#### Pharmacogenomics Research Network Antidepressant Medication Pharmacogenomic Study (PGRN-AMPS)

The PGRN-AMPS is a clinical trial on the response to escitalopram or citalopram of 529 MDD patients over 8 weeks of treatment. The baseline and follow-up assessment of depression severity were performed using the 16-item Quick Inventory of Depressive Symptomatology (QIDS-C16) (40).

#### ISPC Study

The ISPC is an International Consortium established to discover genes that are responsible for SSRIs treatment response in patients with MDD. For our study, we used data from 865 MDD patients recruited in the USA, Germany, Thailand, Taiwan, and Japan who received SSRI treatment. The 17-item Hamilton Depression Rating Scale was used as a measurement tool to assess and follow-up the treatment progress (7).

### Genotyping and Quality Control

The genotype and clinical data for the PGRN-AMPS were available *via* a controlled access system at the database of Genotypes and Phenotypes: dbGaP^1^ and the ISPC data were obtained from the ISPC consortium (7).

For the genotype data of both samples, we implemented quality control (QC) steps using PLINK (41) and samples with low genotype rates <95%, sex inconsistencies (X-chromosome heterozygosity), and genetically related individuals were excluded. We also excluded SNPs that had poor genotyping rate <95%, an ambiguity (A/T and C/G SNPs), a minor allele frequency (MAF ≤ 1%), or showed deviation from Hardy–Weinberg Equilibrium (*p* < 10^–6^).

### Imputations

Genotype data passing QC criteria were imputed in the Michigan server^2^ (42), separately for each study samples using 1000 Genomes project reference panel.

After excluding the low-frequency SNPs (MAF < 10%), poor-quality variants (imputation INFO <0.9 and indels), the imputed dosages were converted to best guess genotypes. The subsequent PGS analyses were performed using the best guess genotypes.

### GWAS Summary Statistics Data

The PGSs were calculated using the approach previously described by the International Schizophrenia Consortium (38). This method requires an estimated effect size for each SNP to compute weighted PGS. The effect estimates (betas) for this study were the summary statistics obtained from previously published GWASs on extraversion, openness, agreeableness, conscientiousness (37), and on neuroticism (35). The data were publicly available for download at http://www.tweelingenregister.org/GPC/ and http://www.thessgac.org/data, respectively. The effect size estimates for each SNP—quantified as beta was extracted from the download file and used to compute weighted PGS in the PGRN-AMPS and ISPC cohorts.

### Definition of SSRI Treatment Outcomes

Treatment response and remission to SSRIs were defined after 4 weeks of treatment follow-up of MDD patients in both cohorts. In addition, PGS associations were evaluated at 8 weeks in PGRN-AMPS. While treatment response was determined as a ≥50% reduction from baseline in the HRSD-17 or QIDS-C16 total scores, SSRI treatment remission was defined as achieving a HRSD-17 score ≤7 or a QIDS-C16 score ≤5 at 4 or 8 weeks of treatment.

Data on the covariates—age, gender, and type of SSRIs medications were also collected and the details can be found in earlier publications (7, 40, 43).

### Statistical Analyses

#### PGS Computation and Association Analyses

**Figure 1:**
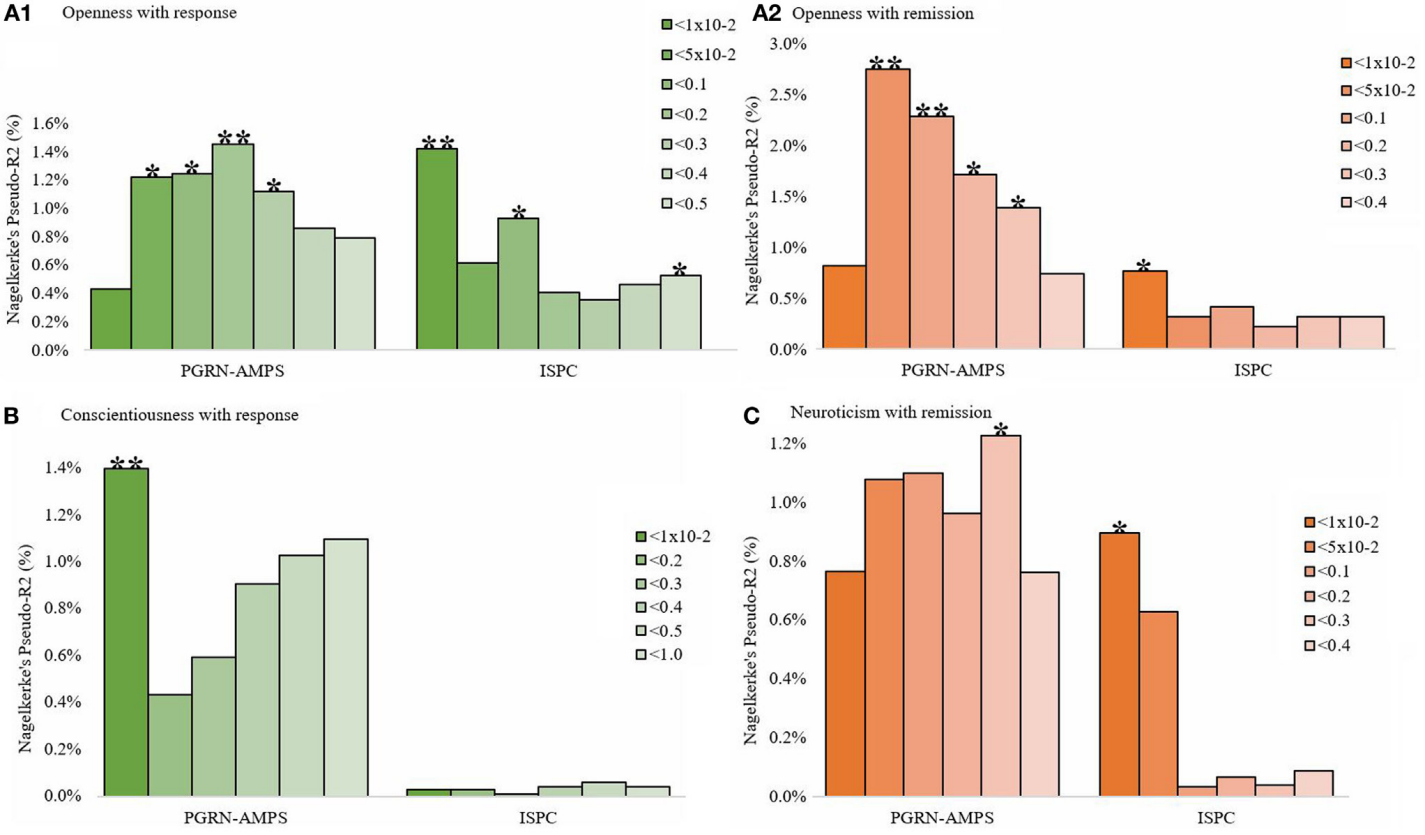
The bar graphs **(A–C)** show the association of the PGSs for the Big Five personality traits with SSRIs response or remission at different *p*-value thresholds (P_T_) after 4 weeks of treatment in the PGRN-AMPS (*n* = 529) and ISPC (*n* = 865) samples. The *y*-axis (Nagelkerke’s Pseudo-*R*^2^) refers to the percentage of variance in SSRIs treatment response/remission accounted for the PGSs of the Big Five personality traits at a particular P_T_ in each sample. On the *x*-axis, plotted from left to right, are the GWAS P_T_ for personality traits used to group the SNPs for the PGSs. The *sign on the top of each bar signify the statistical significance of the PGS association as *p < 0.05, **p < 0.01, ***p < 0.001. Abbreviations: PGRN-AMPS, the Pharmacogenomics Research Network Antidepressant Medication Pharmacogenomic Study; ISPC, the International SSRI Pharmacogenomics Consortium study; SNP, single nucleotide polymorphism; PGS, polygenic score; SSRIs, selective serotonin reuptake inhibitors.

The PGSs were computed for each of the Big Five personality traits using imputed genetic data weighted by GWAS summary statistics of the respective personality traits, separately for the two cohorts: PGRN-AMPS (*n* = 529) and ISPC (*n* = 865) (Table 1; Figure 1). First, quality-controlled SNPs were clumped for linkage disequilibrium (LD) using genome-wide association *p*-value informed clumping with *r*^2^ = 0.1 in a 250-kb window to create an independent SNP-set using PLINK software run on Linux. Next, weighted PGSs were calculated for each individual at a range of *p*-value thresholds (P_T_) as a weighted sum of allele dosages (0, 1, or 2). The P_T_ refers to the *p*-values associated with the effect size of each of the SNPs, as listed in the GWAS summary statistics (35, 37). The weighting was performed by multiplying the dosage of each effect increasing allele by its effect size derived from the GWAS summary statistics (β-coefficient), then divided by the total number of SNPs in each threshold. The PGS was computed at a range of P_T_ (<1 × 10^–2^, <5 × 10^–2^, <0.1, <0.2, <0.3, <0.4, <0.5, and <1.0) separately for each of the two cohorts. Performing the PGS at different P_T_ provides a range of alternative scores to choose the most significantly associated (optimal) PGS that will be used for prediction modeling. At each P_T_, a logistic regression modeling was applied to response/remission to SSRIs (dependent variables) using the PGS for each of the Big Five personality traits as the main predictor variable and adjusting for common covariates, such as age, sex, and cohort-specific covariates including four principal components in the PGRN-AMPS and “study sites” in the ISPC. A statistically significant association between the PGSs for the Big Five personality traits and response/remission to SSRIs was determined at *p* < 0.05, across the P_T_ in both study samples. The prediction accuracy, the percentage of variance explained, Nagelkerke *R*^2^, by the PGSs were calculated as the Nagelkerke *R*^2^ of the full model with PGS and covariates minus the Nagelkerke *R*^2^ of the model with only covariates. To determine the effect of high or low polygenic load on treatment outcomes, the study subjects were grouped into PGS quartiles (Q_1_–Q_4_) at the optimal P_T_. Then, we estimated the odds of treatment response/remission to SSRIs for MDD patients within the group with a high polygenic load for the Big Five personality traits (Q_2_, Q_3_, Q_4_) compared to patients in the lowest PGS quartile (Q_1_).

**Table 1:** Baseline characteristics of major depressive disorder patients and their treatment outcomes with selective serotonin reuptake inhibitors after 4 weeks of follow-up.

Patient characteristics	PGRN-AMPS*N* = 529	ISPC*N* = 865	Total*N* = 1,394
Responders, *N* (%)	206 (44.4)	416 (48.1)	622 (46.8)
Remitters, *N* (%)	128 (27.6)	226 (26.1)	354 (26.7)
Age, mean (SD)	39.6 (13.7)	43.7 (14.7)	42.2 (14.5)
Sex, female, *N* (%)	335 (63.3)	561 (64.9)	896 (64.3)

*PGRN-AMPS, the Pharmacogenomics Research Network Antidepressant Medication Pharmacogenomics Study; ISPC, the International SSRI Pharmacogenomics Consortium study*.

#### Cross-Trait Meta-Analyses of GWASs

In the cross-trait meta-analyses, we applied the O’Brien’s (OB) method and the direct Linear Combination of dependent test statistics (dLC) approach (39, 44, 45), which are implemented in the C^++^ eLX package. Briefly, the OB method and the dLC approach help to combine GWAS effect estimates of genome-wide SNPs, obtained from univariate GWASs and generated two test statistics and associated *p*-values—one for the OB method and one for the dLC method. More details can be found elsewhere (44, 45). The eLX package is available at https://sites.google.com/site/multivariateyihsianghsu/.

Here, GWAS on personality traits that have shown a significant association in the PGS analysis were combined with GWAS on SSRIs treatment outcome. The GWAS summary statistics on SSRIs treatment response (7) were combined with those on (i) conscientiousness (34) and (ii) openness personality (34). Similarly, the GWAS summary statistics on SSRIs treatment remission (7) was meta-analyzed with (i) openness personality (34) and (ii) neuroticism (35).

Statistical significance was determined based on the smaller of the OB or the dLC *p*-values. A significant association was determined if (1) the *p*-value for the cross-trait meta-analysis reached genome-wide significance (*p* < 5 × 10^−8^) and (2) the univariate GWAS effects were at least nominally significant (*p* < 0.05). For each cross-trait meta-analysis, only one lead SNP per locus was reported. Nearby SNPs in LD (*r*^2^ > 0.1) with the lead SNP were considered dependent and belonging to the same locus.

## Results

### Patient Characteristics and Treatment Outcomes

In this study, we analyzed data from 1,394 MDD patients who had SSRI treatment divided into PGRN-AMPS (*n* = 529) and ISPC (*n* = 865) samples. The average age of the patients was 42.2 years and the majority of them (64.3%) were females (Table 1).

Of all patients, 622 (46.8%) were classified as treatment responders with a slight variation across the study samples 44.4% in the PGRN-AMPS and 48.1% in the ISPC. Remission rates were 27.6 and 26.1% in the PGRN-AMPS and ISPC samples, respectively. The rate of remission combined across the two studies was 26.7% (Table 1).

### Association of the PGS for the Big Five Personality Traits with SSRIs Treatment Outcomes

Polygenic scores were computed for each of the Big Five personality traits, and we investigated their association with two SSRI treatment outcomes—response and remission, after 4 weeks (PGRN-AMPS and ISPC) and 8 weeks (PGRN-AMPS) of treatment.

After 4 weeks of treatment, genetic predisposition to openness, conscientiousness, and neuroticism were associated with SSRIs treatment response and/or remission at *p* < 0.05 across PT thresholds, in at least one of the two assessed cohorts (Figures 1A–C). Genetic loading for openness was associated with response and remission in both cohorts (Figure 1A1,2). An elevated PGS for conscientiousness was associated with treatment response, but not remission, in the PGRN-AMPS sample only (Figure 1B). A PGS association for neuroticism with remission, but not treatment response, was shown in both cohorts (Figure 1C). The PGSs for extraversion and agreeableness were associated with neither response nor remission.

We also assessed the level of observed variation in SSRI treatment outcomes accounted for by these personality traits, and found that personality traits at the most significant thresholds explained a considerable amount of variance in treatment outcomes. For example, the PGS for openness accounted for ~1.5% of the observed variation in SSRIs treatment response and ~2.8% of the variance in remission. The PGS for neuroticism explained ~1.5% of the variance in remission and the PGS for conscientiousness contributed to ~1.5% of the variability in SSRI treatment response.

**Figure 2:**
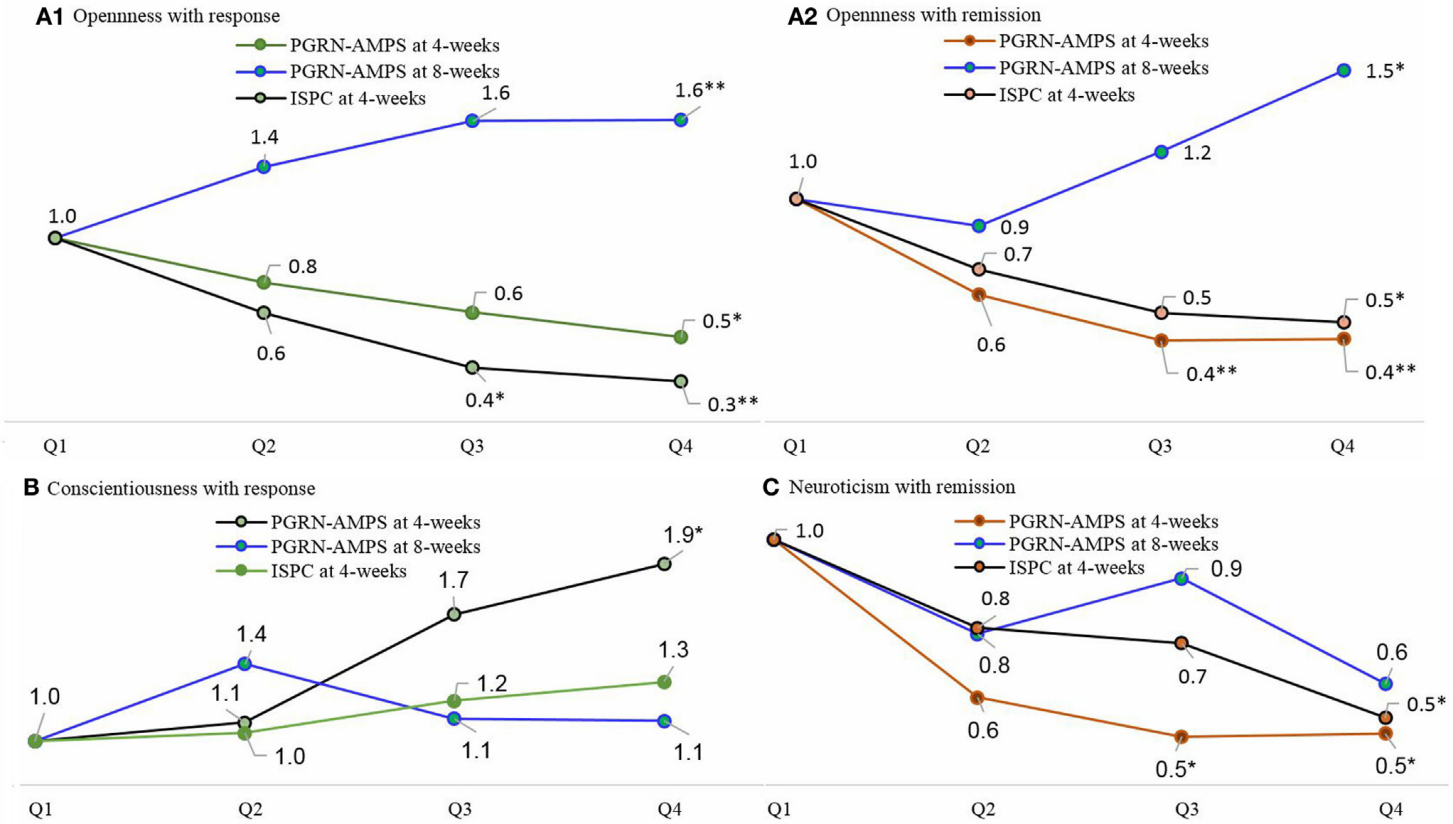
The line plots represent the ORs for favorable response or remission to selective serotonin reuptake inhibitors treatment in patients with MDD with a high personality traits polygenic load (Q2, Q3, and Q4) compared to patients with the lowest polygenic load (Q1), estimated at the most significant *p*-value thresholds. The quartile-based polygenic scores analyses were performed using data at 4 weeks in the ISPC and at 4 and 8 weeks in the PGRN-AMPS. A polygenic loading for openness personality trait was initially associated with poor response and remission to selective serotonin reuptake inhibitors (SSRIs) in the first 4 weeks of treatment (ISPC, PGRN-AMPS at 4 weeks). After a longer (8 weeks) treatment follow-up, the genetic loading for openness had shown a favorable effect to SSRIs response and remission (PGRN-AMPS at 8 weeks). The polygenic loading for conscientiousness personality was favorably associated with response to SSRIs treatment. However, a polygenic loading for neuroticism personality had shown a negative impact on SSRIs remission. The ORs are reported on the lines and the *sign indicates the statistical significance of the ORs as *p < 0.05, **p < 0.01, ***p < 0.001. Abbreviations: PGRN-AMPS, the Pharmacogenomics Research Network Antidepressant Medication Pharmacogenomic Study; ISPC, the International SSRI Pharmacogenomics Consortium study. OR, odds ratio; Q1, quartiles 1; Q2, quartiles 2; Q3, quartiles 3; Q4, quartiles 4; MDD, major depressive disorder.

The status of treatment response and remission for patients in personality trait quartiles (Q2–Q4) was compared with those in the lowest personality trait PGS quartile (Q1) (Figure 2). Our analysis revealed that MDD patients with a high polygenic load for openness personality had initially poorer remission and response rates at 4 weeks of treatment, with Q4 versus Q1 odds ratios (ORs) ranging from 0.30 [ISPC: 95%CI, 0.15–0.59] to 0.52 [PGRN-AMPS: 95%CI, 0.29–0.90] (Figure 2A1,2, green and brown graphs). After longer treatment duration, we observed a reverse effect. Here, a higher polygenic load for openness was associated with a better SSRIs treatment response at 8 weeks in the PGRN-AMPS, with OR of 1.58 [95%CI, 1.10–2.90] (Figure 2A1,2, blue graphs).

Major depressive disorder patients with a higher polygenic load for conscientiousness personality had 1.95 [95% CI, 1.13–3.36] times better SSRIs treatment response compared to those patients in the lowest PGS, although this association was only significant in the PGRN-AMPS sample at 4 weeks of treatment (Figure 2B).

Conversely, MDD patients with a higher polygenic load for neuroticism personality had poorer treatment outcomes with SSRIs. After 4 weeks of treatment, patients in Q4 based on the PGS for neurotic personality had about 50% lower odds of remission compared to patients in Q1 with OR ranging from 0.50 [PGRN-AMPS: 95%CI, 0.28–0.90] to 0.54 [ISPC: 95%CI, 0.33–0.89] (Figure 2C). Constantly, results after 8 weeks of treatment showed a trend inverse association between the PGS for neurotic personality and SSRIs treatment remission, although this was not statistically significant (Figure 2C).

To assess the potential effect of false-positive findings, the association *p*-values were corrected for multiple testing at each P_T_ for SSRIs treatment response and remission using the Benjamini and Hochberg (BH) method. Each of the *p*-values was adjusted assuming a conventionally accepted level of 5% false discovery rate (FDR) (46). After FDR adjustment, the associations of the PGS for openness personality with SSRIs treatment response remained statistically significant (in the ISPC sample: FDR adjusted *p*-value = 0.02 at P_T_ < 1 × 10^−2^) and with remission (in the PGRN-AMPS sample: FDR adjusted *p*-value = 0.04 at P_T_ < 5 × 10^−2^). The PGSs for conscientiousness and neuroticism were not associated with SSRIs treatment outcome after implementing the FDR adjusted *p*-value <0.05.

### Cross-Trait Meta-Analyses of GWASs

**Figure 3:**
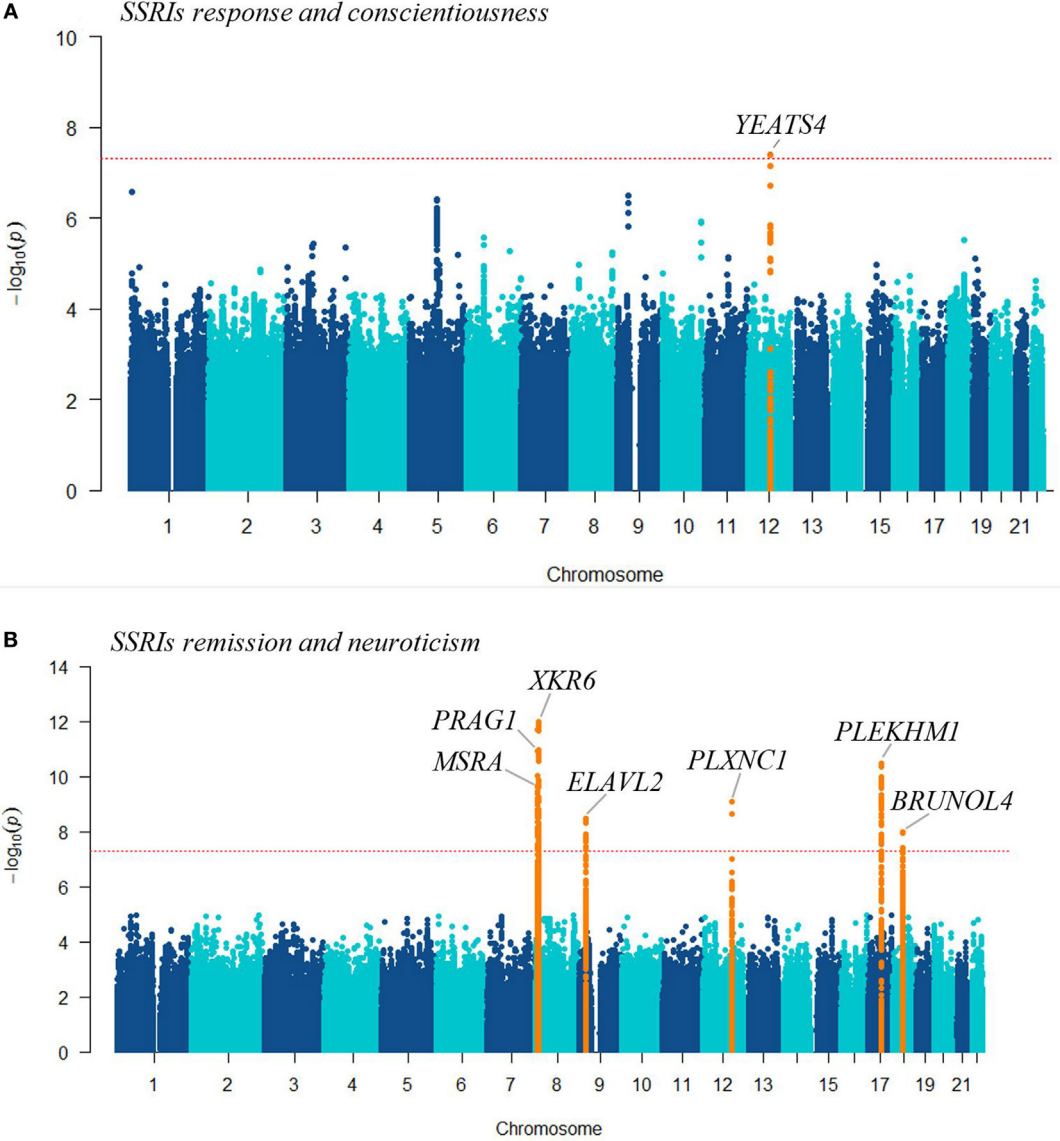
The Manhattan plots show the results of the cross-trait meta-analysis of genome-wide association studies (GWASs) on selective serotonin reuptake inhibitors treatment outcomes (response or remission) with GWASs on: **(A)** conscientiousness personality trait; **(B)** neuroticism personality, highlighting the loci that showed genome-wide significance (orange), and the nearest genes. The −log10 (cross-trait *p*-value) is plotted against the physical position of each SNP on each chromosome. The threshold for genome-wide significance (cross-trait *p*-value <5 × 10^−8^) is indicated by the red dotted horizontal line.

For personality traits that showed a significantly associated PGS, cross-trait GWAS meta-analyses was performed by combining summary GWAS data on SSRIs treatment outcomes and personality traits. Table 2 and Figure 3 summarize the cross-trait meta-analyses findings, including the list of genetic loci and nearest genes that are potentially overlapping between the traits. At a *p*-value of <5 × 10^−8^, we identified eight genetic loci located within or near to protein-coding genes with possible overlapping effects on SSRIs treatment outcomes and personality traits. We found (i) one locus associated with conscientiousness and SSRI response near the *YEATS4* gene (Table 2; Figure 3A) and (ii) seven loci associated with remission and neuroticism located at or near *PRAG1, MSRA, XKR6, ELAVL2, PLXNC1, PLEKHM1*, and *BRUNOL4* genes (Table 2; Figure 3B). From the meta-analyses of SSRIs treatment outcomes with openness personality, we identified only suggestive evidence at significance *p* < 1 × 10^−6^ (Table 2).

**Table 2:** Significant loci resulting from the cross-trait meta-analyses of genome-wide association studies (GWASs) on selective serotonin reuptake inhibitors (SSRIs) treatment response/remission and GWAS on the Big Five personality traits at univariate GWAS *p*-value <5 × 10^−2^ and Cross-trait meta-analysis *p*-value <5 × 10^−8^.

SNP	Chr	Position Ch37	A1	A2	GWAS *p*-value for		Cross-trait GWAS *p*-value	Nearest gene	Effect direction
					SSRIs response (*N* = 865) (7)	Openness (*N* = 260,861) (34)			
rs7555693	1	106838539	A	G	6.46 × 10^−3^	4.485 × 10^−5^	1.37 × 10^−6^	*PRMT6*	−−
rs9321987	6	145030284	A	G	7.49 × 10^−3^	1.056 × 10^−5^	5.05 × 10^−7^	*UTRN*	−−
rs352759	8	15599714	T	A	6.16 × 10^−4^	2.820 × 10^−4^	5.52 × 10^−7^	*TUSC3*	+−
rs7828021	8	50640014	C	G	3.68 × 10^−3^	2.913 × 10^−6^	7.43 × 10^−8^	*SNTG1*	−−
rs11591827	10	82887882	A	G	1.87 × 10^−2^	4.643 × 10^−6^	8.70 × 10^−7^	*SH2D4B*	−−
rs7189979	16	12630187	C	A	2.28 × 10^−3^	1.659 × 10^−5^	1.77 × 10^−7^	*SNX29*	+−
					SSRIs response (*N* = 865) (7)	Conscientiousness (*N* = 260,861) (34)			
rs3825243	12	69750839	A	G	5.78 × 10^−4^	1.41 × 10^−5^	4.04 × 10^−8^	*YEATS4*	−−
					SSRIs remission (*N* = 865) (7)	Neuroticism (*N* = 170,911) (35)			
rs2979204	8	8298857	T	C	3.24 × 10^−3^	5.48 × 10^−10^	8 × 10^−11^	*PRAG1*	++
rs11990063	8	10165195	T	C	4.00 × 10^−2^	6.77 × 10^−9^	9 × 10^−9^	*MSRA*	−−
rs35792458	8	10822431	C	G	1.00 × 10^−2^	5.25 × 10^−10^	1 × 10^−12^	*XKR6*	−+
rs12555870	9	23347724	G	A	4.00 × 10^−2^	1.25 × 10^−6^	1 × 10^−8^	*ELAVL2*	+−
rs4761545	12	94426468	G	T	2.00 × 10^−2^	3.54 × 10^−7^	8 × 10^−10^	*PLXNC1*	++
rs144733372	17	43564222	G	T	1.00 × 10^−2^	1.23 × 10^−9^	3 × 10^−11^	*PLEKHM1*	−+
rs11082011	18	35145122	C	T	1.00 × 10^−2^	8.60 × 10^−9^	4 × 10^−8^	*BRUNOL4*	−+
					SSRIs remission (*N* = 865) (7)	Openness (*N* = 260,861) (34)			
rs55679149	1	89534338	T	C	2.77 × 10^−3^	6.31 × 10^−5^	8.25 × 10^−7^	*GBP1*	+−
rs11728985	4	130036435	T	C	1.93 × 10^−2^	1.24 × 10^−6^	4.03 × 10^−7^	*C4orf33*	−+
rs11155372	6	145019738	T	G	5.11 × 10^−4^	5.85 × 10^−5^	1.28 × 10^−7^	*UTRN*	−+
rs7828021	8	50640014	C	G	1.23 × 10^−2^	2.91 × 10^−6^	4.18 × 10^−7^	*SNTG1*	−−
rs1411216	9	24520194	A	G	7.81 × 10^−3^	6.88 × 10^−6^	4.53 × 10^−7^	*CRIPAK*	++
rs7189979	16	12630187	C	A	2.82 × 10^−3^	1.66 × 10^−5^	2.73 × 10^−7^	*SNX29*	+−

*A1, effect allele; A2, another allele; SNP, single nucleotide polymorphism*.

*The effect direction represents the SNPs effect on SSRIs treatment response or remission for the effect allele based on the ISPC GWAS (7) versus its effect on the GWASs of personality traits as listed in the table*.

## Discussion

In this study, we analyzed data from 1,394 MDD patients who had been treated with SSRIs and assessed whether it is possible to predict antidepressants treatment outcomes—response and remission, using PGS for the Big Five personality traits. To further validate the PGS association findings and provide additional evidence, cross-trait meta-analyses of GWASs on SSRIs treatment outcomes versus GWASs on the Big Five personality traits were performed. Our findings from both analyses found complementary evidence that the association of the Big Five personality traits with SSRIs treatment outcomes is partly genetic.

Among the Big Five personality traits, the PGS for openness, conscientiousness, and neuroticism were significantly associated with SSRI treatment outcomes in patients with MDD. A high polygenic load for openness predicted poorer odds of response and remission to SSRIs after 4 weeks of treatment. However, after 8 weeks of treatment, the odds of response and remission was reversed and high loading for openness was associated with favorable outcomes. Patients with a high polygenic load for conscientiousness had a better odd of response to SSRIs after 4 weeks of treatment, but were neither more nor less likely to have good outcomes after 8 weeks. In contrast, patients who possessed a higher polygenic load for neuroticism risk genetic variants responded poorer to SSRIs treatment at both time points.

The discrepancy between short-term and intermediate-term treatment outcomes in patients with high polygenic loading for openness was unexpected in the context of the previous literature (26, 27), and raises the question whether statements about personality impact on SSRI treatment outcomes can be reliably reached on the basis of assessments conducted within the first month. While longitudinal studies of treatment outcomes in MDD suggest that treatment response within the first month occurs for a majority of patients who will eventually remit (47), they also indicate that there is a considerable proportion of patients who achieve response and remission after much longer treatment periods (48, 49). In this context, our finding raises the possibility that the different Big Five personality traits could have differential effects on early- versus delayed responses to treatment in MDD.

Moreover, the inconsistences in the direction of the relationship between the Big Five personality traits and response to long-term versus short-term treatment to SSRIs might be explained by a psychological theory (50–52). Studies suggested that antidepressants have a primary effect on emotional processing, providing a platform for long-term cognitive and psychological recovery (50), and the clinical effects of antidepressant treatment may be mediated by early changes in emotional processing (51, 52).

In our data, consistency between the outcome parameters—treatment response and remission was variable. Only the PGS for openness showed a significant association with both treatment response and remission. The PGS for conscientiousness was associated with better treatment response, but not with remission. The PGS for neuroticism predicted lower odds of treatment remission, but not poorer treatment response. At face value, these findings suggest that openness and neuroticism could play more important roles in predicting ultimate remission from depressive episodes, whereas conscientiousness might drive early treatment effects rather than longer term outcomes. However, another explanation is that our cohorts might have been underpowered to detect more consistent effects, or that some of the observed associations were chance findings, perhaps driven by multiple testing. Indeed, only the associations of the PGS for openness personality with SSRIs treatment response remained statistically significant after FDR adjustment. Therefore, future genetic studies with higher patients’ numbers are required to confirm our findings.

In all, our genetic findings are in line with previous clinical investigations of the influence of personality characteristics on antidepressant treatment response in MDD. A study in Japan revealed as depressed patients who were resistant to treatment had a higher neuroticism score and lower scores for openness, conscientiousness, and extraversion than patients who remitted and healthy controls (26). In another study, higher clinical scores for openness at baseline were associated with improved treatment response to antidepressants, whereas a higher score for neuroticism was associated with poor treatment outcomes (53). More generally, poor treatment response was associated with personality dysfunction in a large sample study of more than 8,000 antidepressant-treated adults with MDD (27). Similarly, a meta-analysis of 34 clinical studies concluded that MDD patients with a comorbid personality disorder had double the risk of overall poor clinical and treatment outcomes, compared to patients no co-occurring personality disorder (54).

Additionally, previous studies have shown genetic correlations between Big Five personality traits and psychiatric disorders and the PGS for neuroticism was significantly associated with MDD (55).

Since the PGS association reflects a shared genetic etiology, we applied cross-trait GWAS meta-analyses by combining summary statistics on SSRI treatment outcomes with personality traits, and identified eight overlapping genetic loci. The *YEATS4* gene locus was associated with treatment response to SSRIs and conscientiousness. Previously, a gene expression analysis in depressed patients further replicated in mice found lower levels of *YEATS4* in depressed patients compared to healthy controls. Moreover, the expression level of this gene was correlated with the dose of imipramine (a tricyclic antidepressant) (56).

The second gene locus (rs144733372) in *PLEKHM*, which was found in the cross-trait meta-analysis of neuroticism and SSRIs treatment remission, is highly linked (LD: *r*^2^ > 0.8) with several other SNPs located within the *CRHR1* gene. The *CRHR1* gene encodes a G-protein coupled receptor that binds with the neuropeptides of the corticotrophin-releasing hormone family, a major regulator of the hypothalamic–pituitary–adrenal pathway (57). Functional gene polymorphisms in the *CRHR1* gene have been associated with SSRIs treatment response (58), and it moderates the association of maltreatment with neuroticism (59). Corticotrophin-releasing hormone signaling has previously been implicated in mood disorders and treatment response to antidepressants (60).

Another gene showing shared associations with SSRI treatment response and neurotic personality is *MSRA*, which has shown the highest levels of expression in brain tissue (61). Previous studies reported that genetic variants within the *MSRA* gene could be associated with schizophrenia, bipolar disorder (62, 63), executive cognitive function (64), fluid intelligence (63), and self-reported irritable temperament (65).

Further, loci within the *PRAG1* and *PLXNC1* genes have shown overlapping influence on SSRI treatment and neuroticism personality. A genetic polymorphism rs706895C/T within the *FYN* gene belonging to the same family of genes (tyrosine protein kinase family) was significantly associated with personality traits (66). SNPs within the plexin family gene *PLXNA2* have previously been implicated in neuroticism, depression, and psychological distress (67).

Overall, these findings lend further weight to our PGS analyses and reinforce the idea that certain gene polymorphisms have a dual impact on personality structure and antidepressant treatment outcomes in MDD. Studying the individual mechanism of each significant genetic locus in relation to antidepressants in the future studies might lead to novel insights in the molecular underpinnings of these drugs. In conclusion, our study provides evidence in the potential ability of the PGS for the Big Five personality traits to elucidate shared biological mechanisms and to predict SSRI treatment outcomes. Whether these PGSs could be applied to everyday clinical practice in the future relies on their ability to stratify MDD patients into categories of good treatment responders versus nonresponders. Further research is required to determine if this is the case. However, the small effect sizes found in our study give rise to cautious interpretation. In our view, their full clinical value likely lies in their contribution to multi-variable models that also comprise clinical and environmental factors influencing medication response.

## Author Contributions

AA, KS, and BB developed the study concept and design, performed the statistical analysis, and drafted the manuscript. Other authors contributed data, resources and were involved in critical revision of the manuscript, obtained funding, and contributed to cohort and genetic data and study supervision.

## Conflict of Interest Statement

The authors declare that the research was conducted in the absence of any commercial or financial relationships that could be construed as a potential conflict of interest.

## Footnotes

^1^http://www.ncbi.nlm.nih.gov/gap.

^2^https://imputationserver.sph.umich.edu
